# Humins Blending in Thermoreversible Diels–Alder Networks for Stiffness Tuning and Enhanced Healing Performance for Soft Robotics

**DOI:** 10.3390/polym14091657

**Published:** 2022-04-20

**Authors:** Kenneth Cerdan, Joost Brancart, Ellen Roels, Bram Vanderborght, Peter Van Puyvelde

**Affiliations:** 1 Department of Chemical Engineering, Soft Matter, Rheology and Technology (SMaRT), KU Leuven, Celestijnenlaan 200J, 3001 Heverlee, Belgium; peter.vanpuyvelde@kuleuven.be; 2 Physical Chemistry and Polymer Science (FYSC), Vrije Universiteit Brussel, Pleinlaan 2, 1050 Brussels, Belgium; joost.brancart@vub.be; 3 Brubotics and Imec, Vrije Universiteit Brussel, Pleinlaan 2, 1050 Brussels, Belgium; ellen.roels@vub.be (E.R.); bram.vanderborght@vub.be (B.V.)

**Keywords:** humins, self-healing, Diels–Alder, mild healing, biomass valorization, green composites, immiscible blends, stimuli-responsive, thermoreversible, soft robotics

## Abstract

Humins waste valorization is considered to be an essential pathway to improve the economic viability of many biorefinery processes and further promote their circularity by avoiding waste formation. In this research, the incorporation of humins in a Diels–Alder (DA) polymer network based on furan-maleimide thermoreversible crosslinks was studied. A considerable enhancement of the healing efficiency was observed by just healing for 1 h at 60 °C at the expense of a reduction of the material mechanical properties, while the unfilled material showed no healing under the same conditions. Nevertheless, the thermal healing step favored the irreversible humins polycondensation, thus strengthening the material while keeping the enhanced healing performance. Our hypothesis states a synergistic healing mechanism based on humins flowing throughout the damage, followed by thermal humins crosslinking during the healing trigger, together with DA thermoreversible bonds recombination. A multi-material soft robotic gripper was manufactured out of the proposed material, showing not only improved recovery of the functional performance upon healing but also stiffness-tunable features by means of humins thermal crosslinking. For the first time, both damage healing and zone reinforcement for further damage prevention are achieved in a single intrinsic self-healing system.

## 1. Introduction

The sustainable production of organic chemicals, fuels, and materials that are able to replace the classic fossil fuel-derived analogues has been the main motivation of many biorefinery technologies. More precisely, the development of efficient bio-based plastics offers an attractive opportunity to substitute their petro-based counterparts using renewable resources [1]. An example of such materials is the so-called polyethylene furanoate (PEF), developed by Avantium Renewable Polymers BV, which satisfies the requirements to be a suitable candidate to complement, and eventually substitute, polyethylene terephthalate (PET) in both price and performance [2]. PEF synthesis involves the acid treatment of saccharides to obtain the platform molecule 2,5-furandicarboxylic acid (FDCA), followed by its polycondensation with glycols [3]. However, one of the main drawbacks of the acid-catalyzed processing of biomass-derived sugars is the unavoidable humins formation. Humins are a black, viscous, heterogeneous, highly polydisperse macromolecular side-product that arise from polycondensation reactions of sugar-derived furanic monomeric units, affecting the overall yield and economic viability of the process [4].

Researchers have worked intensively to understand the formation path and molecular structure of humins [5,6] and find strategies to suppress their production during the biorefinery process [7]. In addition, novel strategies towards their valorization have been explored, either via chemical treatment to obtain valuable platform molecules [8,9,10] or via the development of humins-derived materials. The latter has recently shown promising outcomes for the preparation and application of a wide variety of different materials, including thermoset resins [11,12,13], elastomers [14], bio-foams [15,16], and fibers [17]. Interest in the utilization of humins to obtain high added-value products is expected to grow as their valorization will encourage the circularity of biorefinery operations towards a zero-waste process. Despite the fact that the development of novel economically viable bio-based material formulations is a promising valorization route, either an appropriate degradation and/or recycling method or a way to extend these products life span is mandatory to avoid their landfilling or release to the environment after usage [1].

In this regard, self-healing (SH) polymers arise as an attractive approach to extend the service lifetime of plastics. These materials have the capability to repair their own structure after micro- or macroscopic mechanical damage is applied, avoiding material failure and recovering their initial mechanical performance [18]. One of the most extended classification criteria of SH materials is based on the healing mechanism. Hence, SH polymers can repair damage either by exhaustion of healing agents that are stored within microcapsules [19] or vascular networks [20] in a polymeric matrix, so-called extrinsic SH materials. Alternatively, healing can be done by the restoration of reversible links (supramolecular interactions or dynamic covalent bonds) inherent to the material chemical nature, known as intrinsic SH materials [21]. Extrinsic materials can heal autonomously under ambient conditions but only for a limited amount of healing cycles at the same damage location, as the healing agent is irreversibly consumed after the damage repair. On the other hand, intrinsic materials can, theoretically, be healed an infinite number of times, provided that the chemical nature remains unaltered. However, in most cases, an external trigger (heat [22], light [23,24], moisture [25], etc.) is required to enhance molecular diffusion enough to close the damage gap, bringing the reversible bonds back in contact and promoting the rearrangement of the matrix. SH polymers have found very promising applications in coatings [24,26], sensors [27], or robotics [28,29,30]. More precisely, the SH soft robotics upsurge has incited novel trends to find more eco-friendly solutions, as well as to enhance their performance and capabilities [31,32].

During the last decade, substantial progress has been made to divert conventional petroleum-based SH materials to more sustainable, bio-based alternatives [33]. The combination of natural polymers such as cellulose [34], alginate [35], chitosan [36], or lignin [37,38,39] with the required chemical modifications to provide healing ability have inspired an extensive variety of materials that orchestrate high properties versatility by tailoring their chemical structure design. Nevertheless, these materials (especially intrinsic SH materials) possess a relatively complex preparation process and require harsh external triggers and long healing timescales to accomplish an efficient repaired state [33].

To address these challenges, we report for the first time a polymer composite prepared by blending humins with a healable material based on thermoreversible Diels–Alder (DA) crosslinks using a furan-maleimide system. DA thermoreversible polymer networks and composites exhibit excellent healing performance by making use of temperature, often needing highly energetic thermal treatments for a complete material recovery [40]. Incorporation of viscous liquids within an elastomeric polymer matrix can provide a subsequent plasticizing effect. Plasticizers are known to increase the polymer chain mobility, thus enhancing the healing performance of intrinsic SH materials at the expense of the mechanical properties. However, humins can not only exhibit this plasticizing effect on the DA matrix, but also undergo irreversible intramolecular crosslinking via condensation reactions when exposed to moderate temperatures, hence influencing the final material stiffness and mechanical strength upon thermal postprocessing. The resulting stiffness-tunable material shows interesting properties to be further exploited as a new source for the preparation of SH soft robotics. To keep the mechanical properties of the actuator consistent when submitted to a healing thermal treatment, focalized healing using a welder is proposed to expose only the damaged zone to a thermal source, efficiently closing the cut and subsequently reinforcing the healed spot, thus preventing any potential future damage.

## 2. Materials and Methods

### 2.1. Materials

Humins were kindly provided by Avantium Renewable Chemicals BV and used as received. The polyetheramine Jeffamine D400 with an average molecular weight of 484 g·mol^−1^ was kindly provided by Huntsman (Everberg, Belgium). Furfuryl Glycidyl Ether (FGE) was purchased from Acros Organics (Geel, Belgium). A low-viscous aliphatic bismaleimide (BMI689) was purchased from Caplinq (Assendelft, The Netherlands).

### 2.2. Synthesis of BMI689-F400 and Humins-DA

A facile synthesis protocol was followed to prepare the proposed material. First, humins were preheated at 90 °C for 15 min to reduce their viscosity, followed by the addition of a furan-functionalized diamine (Jeffamine D400 reacted with FGE following a previously described synthesis procedure [41]) named F400. Then, the mixture was reacted with a low-viscous aliphatic bismaleimide BMI689 using stoichiometric amounts of furan and maleimide functional groups. The mixture was centrifugally mixed for 5 min and the resulting viscous liquid was poured in stainless steel molds for compression molding at room temperature. Plates and bar molds were used for rheological and mechanical characterization, respectively. The process led to the polymerization of the final DA crosslinked composite, namely Humins-DA. A humins:DA ratio of 30:70 was chosen for this study, following qualitative healing assessments. This proportion was chosen as it is the minimum amount of viscous liquid that exhibits an influence on the wetting behavior of the damage area in the composite at room temperature, without becoming too sticky due to a high humins loading.

### 2.3. Characterization and Self-Healing Efficiency Determination

#### 2.3.1. ATR-FTIR Spectroscopy

The composition of humins and Humins-DA was studied using a Thermo Fisher Scientific Nicolet 6700 FT-IR spectrometer (Thermo Fischer Scientific, Merelbeke, Belgium). Signals were recorded in single bounce Attenuated Total Reflectance (ATR) using a Nicole smart iTR accessory with a diamond-coated ZnSe crystal. The analysis was performed at room temperature, previously correcting atmospheric contributions via measuring and subtracting the background signal.

#### 2.3.2. Thermal Analysis

The thermal stability was studied by Thermogravimetric Analysis (TGA) using a Q500 TGA (TA Instruments, Antwerp, Belgium) performing a heating ramp of 10 K min^−1^ up to 550 °C under N_2_ atmosphere at a purge flow of 10 mL·min^−1^. The sample mass of the analyzed materials ranged from 5 to 15 mg. The influence of the presence of humins on the glass transition temperature was evaluated by Differential Scanning Calorimetry (DSC) using a Q2000 DSC (TA Instruments, Antwerp, Belgium) equipped with a Refrigerated Cooling System (RCS). A N_2_ gas purge flow of 25 mL·min^−1^ was used to purge the DSC cell. For this study, several heating/cooling cycles from −80 to 50 °C at a rate of 5 K·min^−1^ were applied to 5 to 10 mg samples loaded in non-hermetical Tzero Al crucibles. A maximum temperature of 50 °C was chosen to keep the humins’ chemical integrity during the experiment.

#### 2.3.3. Scanning Electron Microscopy

The structural morphology of the Humins-DA material was observed by using a JEOL JSM-6010LM SEM, operating at 15 kV accelerating voltage. Prior to visualization, all samples were sputter-coated with a thin Au/Pd layer using a JEOL JFC-1300 autofine coater (JEOL, Lireweg, The Netherlands) to minimize sample charging during imaging.

#### 2.3.4. Rheological and Mechanical Analysis

The viscoelastic and mechanical properties were investigated by Dynamic Rheology and Dynamic Mechanical Analysis (DMA), respectively. Rheological experiments were performed on an AR-G2 rheometer (TA Instruments, Antwerp, Belgium). The rheometer was equipped with a convection oven as a heating system using air atmosphere and a stainless steel 25 mm parallel plate geometry. Linear dynamic oscillatory experiments were carried out under both non-isothermal and isothermal conditions to evaluate the gel transition and the thermal stability at a certain temperature, respectively. The gel transition temperature (T_gel_) is determined as the cross-point where the phase angle becomes frequency independent, hence five discrete frequencies following a logarithmic distribution were used for its determination under a heating ramp of 1 K·min^−1^. For the isothermal experiments, a single frequency of 1 Hz was used at a constant temperature of 60 °C. A strain of 1% was used for both experiments to perform measurements in the linear viscoelastic region. Second, DMA tests were performed on a Q800 DMA (TA Instruments, Antwerp, Belgium) under tension mode. Stress-strain tests until fracture were performed at room temperature by subjecting rectangular specimens to tensile strains with a strain rate of 60%·min^−1^. The Young’s modulus was calculated as the slope of the stress-strain curve in the linear region at low deformations (0–0.5%). Finally, healing experiments were conducted for the neat, unfilled, and humins-filled DA polymer networks. To determine the healing efficiency, the Young’s modulus and the stress and strain at fracture were compared before and after the healing process, which was triggered by submitting the sample to an isothermal step of one hour at either 60 or 80 °C.

### 2.4. Humins-DA Reprocessing via Extrusion

Humins-DA were reprocessed using a mini extruder (DSM Xplore, Xplore, Sittard, The Netherlands) recirculating 5 cm^3^ of material with two co-rotating 150 mm screws. Samples loaded were mixed for 5 min at 100 °C, at a constant screw rotation speed of 100 rpm. Afterwards, the resultant mix was poured on stainless steel rectangular molds for further mechanical testing.

### 2.5. Characterization of the Soft Robotic Actuator

The soft tendon-driven fingers developed in this work were characterized using an in-house developed test setup. The finger bending characterization setup consisted of a Raspberry Pi 4 (Raspberry Pi, UK) that was connected to a camera and an Arduino Uno. The Arduino controlled a stepper motor that spooled the tendon wire using a TMC2130 driver. It communicated the angle of the motor to the Raspberry Pi, such that the tendon length could be calculated. Meanwhile, the camera tracked the position of the finger. To this end, the finger under test was fitted with five white markers on the side of the backbone, at both sides of each phalanx. The position of each marker in an image was recorded in Matlab during postprocessing of the data. The bending performance of the finger was assessed by applying a sinusoidal motion on the tendon length.

## 3. Results and Discussion

### 3.1. Reactants Employed and Thermal Properties of the Synthesized Materials

**Figure 1:**
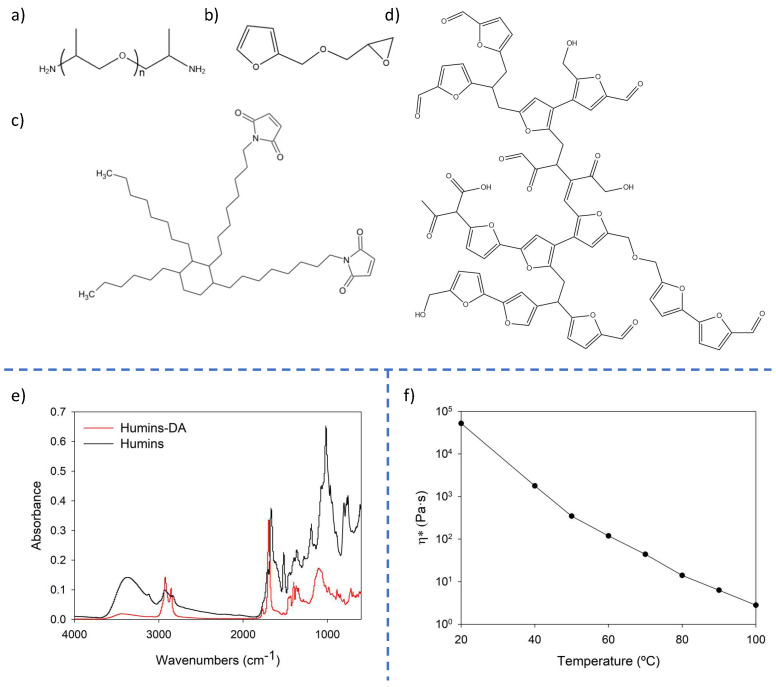
Chemical structures of the reactives employed: (**a**) Jeffamine D400, (**b**) Furfuryl Glycidyl Ether, (**c**) BMI689, (**d**) humins (adapted from [5]), (**e**) FT-IR spectra of Humins-DA and raw humins, and (**f**) humins complex viscosity modulus variation as a function of temperature.

The chemical structures of the reagents employed for the synthesis are given in Figure 1. Figure 1d provides an accepted representation of the chemical structure of humins, bearing many furan groups with different substituents. The infrared spectrum of the humins (Figure 1e) is similar to those reported in literature [14,15]. The most important peaks are assigned and summarized in Table 1. Both a high concentration of oxygenated functional groups, and an elevated proportion of unsaturations, were observed for humins composition. First, efforts were made to accomplish the straight DA reaction between the humins and the bismaleimide BMI689; however, the DA reactivity of the humins furan groups was too low to create a covalently crosslinked gel. It is well-known that electron-withdrawing substituents on the furan rings lower the reactivity towards the DA reaction [42]. The humins’ high chemical complexity, as well as the phase separation ensuing the bad miscibility of the two compounds, yielded a broad IR absorption which made monitoring this process via FT-IR not possible. For these reasons, another approach was taken in which the humins were blended with a furan-functionalized Jeffamine F400 and bismaleimide BMI689. The humins were mixed with the reactive compounds and the polymer network was formed in the presence of the humins. The final material can be considered as a semi-interpenetrating polymer network.

An important question when developing Humins-DA materials is the choice of the optimal amount of humins. Proportions corresponding to 40:60 and 20:80 humins:DA ratios were also evaluated during the blend composition optimization process. On one hand, a 40:60 composition led to a sample overfilled of humins, being impossible to be properly handled. This occurred as a consequence of the sample high stickiness and very poor mechanical properties, even losing the morphology of the manufactured piece when manipulated. On the other hand, a 20:80 proportion exhibited a very poor dispersion quality of humins within the DA matrix, showing even by glance certain regions of the composite not filled with humins as a consequence of bad miscibility. In addition, lower humins loadings entailed a higher humins confinement within the highly crosslinked network, limiting their flow and not showing any visible difference in the healing behavior as compared to BMI689-F400. As a compromise, 30 wt% of humins were blended into the BMI689-F400 DA polymer network. Dynamic rheometry measurements were performed to assess the viscosity and viscoelastic response of the pure humins under different temperatures that could be suitable conditions as the thermal healing trigger (Figure 1f). The modulus of the complex viscosity (η*) changed four orders of magnitude from 10^5^ to 10^1^ Pa.s when heated stepwise from 20 to 100 °C.

**Figure 2:**
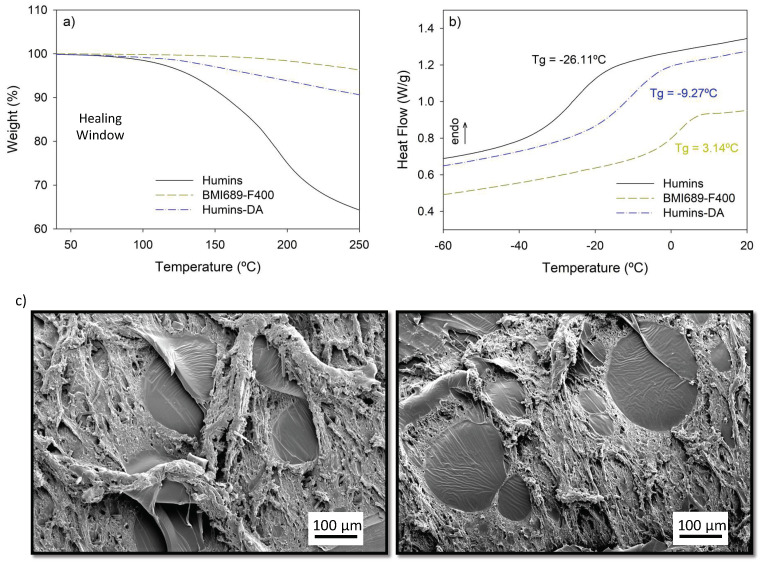
(**a**) TGA results of humins (black), BMI689-F400 (dark yellow) and Humins-DA blend (blue) at 5 K·min^−1^, (**b**) DSC analysis of humins (black), BMI689-F400 (dark yellow) and Humins-DA (blue). Samples were cooled to −80 °C at 5 K·min^−1^ and re-heated at 5 K·min^−1^, (**c**) SEM pictures of the Humins-DA cross-section.

The proposed thermoreversible product requires a sufficiently high thermal stability to prevent material degradation during the healing process and during thermal (re)processing. Based on previous research, it is expected that temperatures higher than 100 °C are not needed to either achieve a fully healed state [43] or for most commonly applied thermal processing techniques on these DA-based polymer networks [44]. TGA experiments show no significant weight losses for the neat polymer matrix and the Humins-DA blend within the expected healing and (re)processing temperature range up to at least 100 °C (Figure 2a). Thus, temperatures up to 100 °C were chosen as potential healing temperatures to avoid any mass loss during the application and reprocessing of the material. The raw humins showed lower thermal stability, as a small weight loss was observed already at temperatures from 100 °C onward, suggesting gradual loss of small molecule fractions. While the weight loss was drastically reduced when confined in the reversible polymer network, special attention needs to be given to long exposures times at high temperatures, as the release of volatiles could lead to porosity generation and variations in the composite mechanical strength. Thus, a temperature of 60 °C (η* ≈ 10^2^ Pa.s) was chosen in this research as a potential candidate to substantially lower the viscosity of humins while keeping their chemical nature as unaltered as possible and to thermodynamically favor the formation of the DA adducts. Under the proposed low healing temperature, low reaction medium mobility, low humins’ furans reactivity, and absence of a catalyst, it was not expected that any humins functionalities could side react with the proposed F400 and BMI689 monomers. To corroborate this assumption, FT-IR analysis of Humins-DA was performed, and the BMI689-F400 component was subtracted and compared to the raw BMI689-F400 polymer, showing no significant differences (See Figure S1, from Supplementary Materials). DSC measurements (Figure 2b) show the influence of humins on the glass transition temperature (T_g_) of the formed blend, analyzed by performing a heating/cooling cycle from −80 to 50 °C. A plasticizing effect was observed when adding humins with a low T_g_ of around −26 °C to a polymer network with a higher T_g_. The incorporation of humins reduced the T_g_ from 3.1 to −9.3 °C, enhancing polymer chain mobility. The single glass transition for Humins-DA implies that a homogeneous system was obtained by good mixing of the humins and the DA network.

Compatibility and phase morphology are crucial aspects when preparing polymer blends. In contrast to the DSC measurements, phase separation was observed using SEM (Figure 2c), showing a droplet-matrix immiscible polymer blend morphology. Humins droplets could be observed throughout the DA matrix with a range of droplet diameters from submicron size up to several hundred μm. The humins were highly aromatic and polar, while BMI689 and F400 were aliphatic. The bismaleimide BMI689 was mostly apolar, while the furan-functionalized Jeffamine F400 was rather polar and expected to be somewhat compatible with the humins due to the polarity and presence of the furan molecules. The single T_g_ observed in DSC (Figure 2b) was not in good agreement with the phase morphology observed in SEM. Since the humins droplet size confined within the DA network was big enough, it would be expected to observe to different T_g_, one for each component. The glass transition temperatures of both pure phases were only about 30 °C apart. Partial mixing of one phase into the other will induce a shift of the glass transitions of the two phases, possibly resulting in a single, broad transition. It was not possible to differentiate two transitions using the derivative of the heat flow signal to confirm this hypothesis. To the authors’ knowledge, this research shows for the first time the preparation of an immiscible polymer blend using humins.

### 3.2. Rheological Behaviour during the Gel Transition

**Figure 3:**
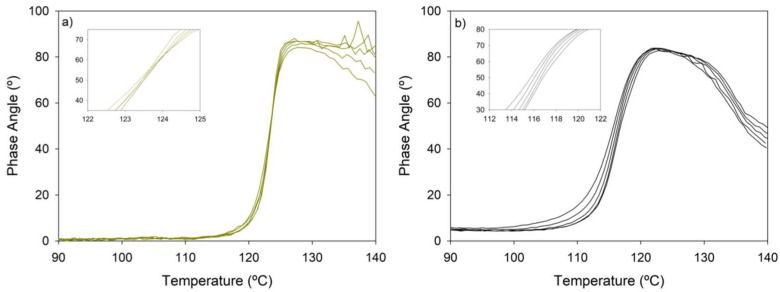
Dynamic rheology measurements at a heating ramp of 1 °C·min^−1^ to determine the degelation of (**a**) BMI689-F400 and (**b**) Humins-DA. Frequencies (Hz): 0.3; 0.6; 1; 1.8; 3.1 (one curve for each frequency). A Tgel value of 123 °C is found for BMI689-F400 whereas no clear T_gel_ was found for Humins-DA despite the gel transition being shifted to lower temperatures.

The influence of humins on the gelation behavior was derived using dynamic rheology. The gel transition temperature (T_gel_), going from predominantly solid, elastic behavior to viscous, liquid-like behavior was determined according to Winter’s criteria as the point where the phase angle becomes frequency independent in a multi-frequency experiment [45]. For BMI689-F400, a T_gel_ value of 123 °C was found (Figure 3a), whereas for the humins composite, no cross-point was observed. Still, a clear degelation transition of the polymer network could be observed as the phase angle increased for all the frequencies up to 80°, demonstrating clear viscous, liquid-like behavior (Figure 3b). Moreover, a significant decrease in the phase angle was visible at temperatures well above the T_gel_, which suggests an increase in the elastic behavior of the viscoelastic composite. This phenomenon could find its origin in either of three effects taking place during degelation or a combination thereof. First, humins can self-crosslink via post-condensation reactions under the studied thermal conditions, which is translated into an increase in humins M_w_ that enhances the elastic performance of the composite. The thermal behavior of humins has been thoroughly investigated, reflecting the same curing behavior of humins on their viscoelastic properties, being able to reach T_g_ values up to 80 °C at total reaction conversion [15,46,47,48,49]. This could bring some issues in terms of material thermal reprocessability as the internal structure of the composite can be influenced by extensive thermal treatments [50]. Second, this phase angle behavior was previously found for other composites based on thermally reversible polymer networks, for which it is hypothesized that the interactions between the solid particles forms a filler network, limiting the mobility of the unbonded furan and maleimide monomers in their liquid state [51]. The large polydispersity of humins involves not only small liquid molecules but also the presence of solid, high M_w_ macromolecules which can also hinder the monomer mobility, holding the main role of the rheological properties above the gel transition. Finally, a minor weight loss was observed at temperatures above 100 °C for the pure humins and the composite, matching the region where the phase angles of the dynamic rheology experiment decreased. This mass loss of small molecules could entail an increasing average M_w_ of the humins in the high temperature region of the experiment, where the material is in the liquid state. Furthermore, the incorporation of humins shifted the gel transition towards lower temperatures, due to the decreased concentration of furan and maleimide adduct bonds in the composite, shifting the Diels–Alder reaction equilibrium.

### 3.3. Mechanical Properties and Self-Healing Efficiency

DMA experiments were performed to determine the mechanical properties of Humins-DA and compared to the pure BMI689-F400 network. The parameters studied were the fracture stress (σ), fracture strain (ε), and Young’s modulus (E) (Table 2). First, it should be noted that the presence of humins reduced the fracture stress and Young’s modulus, while the fracture strain remained nearly unchanged. These results are in line with the plasticizing effect of the humins. It is hypothesized that, due to the high polydispersity of humins, the influence of big macromolecular clusters in terms of chain mobility when deformation is applied hinders the larger elasticity provided by the small molecules that act as plasticizers. Immiscibility of the phases can also induce interfacial slippage upon force application, thus diminishing sample yielding at large deformations due to the weak applied force transmission into the dispersed minor phase. To quantitatively appraise the healing efficiency of the proposed materials, sample specimens were cut in two pieces with a scalpel, and the cut parts were manually brought back into contact, commencing the healing action. Then, the samples were heated at 60 °C for 1 h, followed by a slow cooling to room temperature to restore the Diels–Alder equilibrium and, consequently, the crosslink density and mechanical properties. The healed materials were submitted to the same tensile testing conditions and the fracture properties were compared. The healing efficiency (η) is calculated as the ratio between the material property after healing and before damage. A clear healing performance enhancement was observed for Humins-DA, recovering an important part (roughly 80%) of its initial properties, while the original counterpart BMI689-F400 did not exhibit any healing under the same mild healing conditions. In addition, after applying such a healing process, an increase in the Young’s modulus of Humins-DA was observed as compared to BMI689-F400, showing a non-representative healing efficiency of 177%. This phenomenon is not realistic and can be attributed to intramolecular crosslinking of humins under such conditions within the polymeric matrix, translated into a stiffening development. Thus, humins post-condensation during the healing process, as well as during processing, could influence the material properties when exposing it to external thermal sources for successive times such as lower healing efficiencies due to humins restricted mobility upon crosslinking and mechanical properties strengthening.

### 3.4. Stiffening Effect upon Heating and Healing Mechanism Elucidation

**Figure 4:**
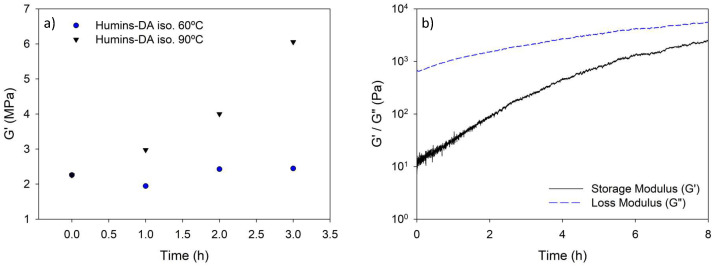
Isothermal small amplitude oscillatory time sweeps at 60 °C for (**a**) Humins-DA and (**b**) humins. Both tests were performed at a frequency of 1 Hz and an amplitude of 1%.

To gain insights into the stiffening effect observed during the thermal healing treatment, dynamic rheometry measurements were performed on the Humins-DA. An isothermal oscillatory time sweep was performed at 60 °C for several hours to monitor the changes in the viscoelastic properties. The results in Figure 4a show no noticeable changes at short timescales, representative for the healing procedure. This is not in agreement with the DMA results obtained. Nevertheless, increasing the temperature to 90 °C demonstrated a noticeable difference in the storage modulus G’ value of Humins-DA. Subsequently, an oscillatory time sweep was performed on the raw humins at 60 °C to isolate the influence of temperature on their rheological behavior to be extrapolated to Humins-DA properties, i.e., without the influence of the elastic DA matrix on the thermo-rheological behavior. In this case, the results obtained (Figure 4b) displayed a steady increase of the viscoelastic response, even at short timescales where the healing experiments were occurring. The observed increase confirmed the hypothesis that humins intramolecular crosslinking influences the mechanical properties of Humins-DA upon healing, despite the fact that these changes are not visible for the composite rheology test.

**Figure 5:**
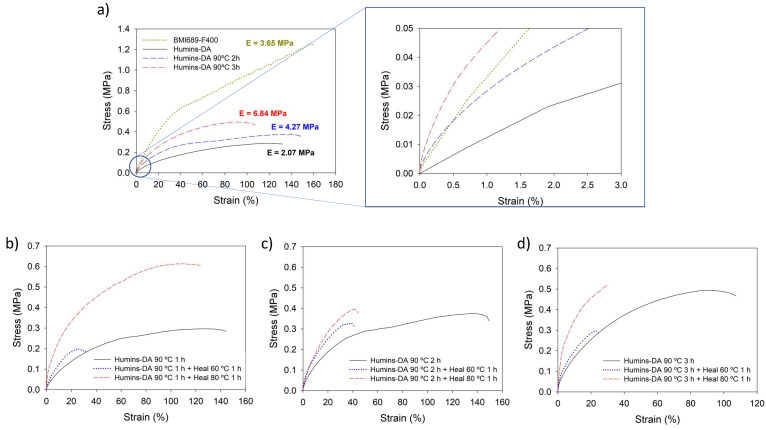
(**a**) Fracture tests for BMI689-F400 (yellow dotted line), Humins-DA as synthesized (black solid line), Humins-DA treated isothermally at 90 °C for two hours (blue dashed line) and three hours (red dash-dotted line). Fracture tests for Humins-DA (black line), Humins-DA healed at 60 °C for 1 h (blue dotted line), and Humins-DA healed at 80 °C for 1 h (red dashed line) when treated isothermally at 90 °C for (**b**) one hour, (**c**) two hours, and (**d**) three hours.

To elucidate the thermal stiffening influence on Humins-DA mechanical properties, fracture tests were performed on samples treated isothermally at 90 °C for increasing isothermal times (Figure 5a). Averaged mechanical properties parameters of the thermally annealed samples, together with the respective healing efficiencies at both 60 and 80 °C for 1 h, are summarized in Table 3. As previously observed, there is a clear plasticizing effect upon incorporation of humins in the Humins-DA hybrid system. However, this can be partially compensated by thermally treating the sample, in this case at 90 °C. An enhancement of the Young’s modulus of Humins-DA from 2.46 to 4.38 MPa and 5.41 MPa was observed when heated at 90 °C for two and three hours, respectively. In addition, there was a partial compensation of the lost mechanical strength. The resulting material stiffness became higher than BMI689-F400 with a Young’s Modulus of 3.66 MPa. This stiffening effect emerged as a consequence of humins crosslinking within the DA network, increasing their M_w_. Longer thermal exposures entailed both a growth of fracture stress as well as a decrease in fracture strain. Thus, humins crosslinking exhibited a stiffening effect on the formed composite when thermally treated at the expense of the stretchability, since high M_w_ humins fractions also acted as stress concentration points, making the binary composite system easier to be fractured. Hence, the humins served as a reinforcing filler due to the formation of bigger crosslinked macromolecular clusters, instead of a plasticizer. The stiffening effect of the humins on the composite also affected the self-healing properties. Figure 5b exhibits the stress-strain curves of Humins-DA treated at 90 °C for 1 h. Samples healed for 1 h at 60 °C (blue) and 80 °C (red) were compared to the original stress-strain behavior (black). The healing efficiency at 60 °C of the thermally treated material drastically decreased compared to the untreated sample, as the plasticizing effect of the humins decreased. Healing at 80 °C for 1 h showed much higher mechanical properties recovery, resulting in a total strain recovery and a great development of the mechanical strength of the sample following further thermal treatment during the healing cycle at a more elevated temperature. Hence, the thermal treatment of the humins resulted not only in a stiffening effect on Humins-DA at small deformations but also entailed a great stress recovery of the sample properties. For the samples submitted to longer thermal treatments of two hours (Figure 5c) and three hours (Figure 5d), further mechanical strengthening was observed, as discussed previously. When the samples were healed, the recovery of the strain at break was limited as a consequence of the loss of plasticizing effect of the humins. Conversely, the stress at break was recovered completely after 1 h at 80 °C. Finally, no visible variations in the humins droplet morphology were observed when Humins-DA was submitted to a prolonged thermal treatment (See Figure S2, from Supplementary Materials).

The obtained results demonstrate that the humins play a crucial role, not only on the mechanical properties, but also on the Humins-DA healing performance. We hypothesize that there are synergistic mechanisms between humins and the DA crosslinked polymer that improve the healing behavior of the composite compared to the pure DA polymer network. This effect consists of the combination of two healing mechanisms in a single SH system: intermolecular diffusion carried out by humins and dynamic covalent bonds rearrangement derived from the DA network. Essentially, once a damage is applied to the material containing the humins, the viscous liquid components of humins are able to diffuse throughout the damage and enhance the wetting of the broken surfaces. This diffusive flow induces the mobility required by the DA system to bring the furan and maleimide moieties back in contact, which is normally achieved under the application of heat. Under the proposed conditions, as the DA reaction is thermodynamically favored at low temperatures, healing is efficient even under a mild thermal treatment if the surface contact is close enough. Due to humins high viscosity under ambient conditions, their incorporation is possible at high proportions (30 wt% in this research), while keeping high enough mechanical properties. Alternatively, it could be hypothesized that the healing mechanism first consists of a gluing effect of the humins followed by a post-condensation step. As humins are highly viscous at room temperature, the broken surfaces stick to each other upon contacting, due to the humins acting as a glue. Upon heating at 60 °C, humins can condense and increase the mechanical strength of the composite, both within the matrix and at the crack surface. Thus, a healing effect would be detected which is not directly related to DA bonds recombination but to humins diffusive recombination followed by thermal condensation. However, it can be argued that the viscosity of humins is not high enough to obtain the observed properties in the DMA results even after a mild thermal treatment and consequent condensation. Especially, the increase of the Young’s modulus and the high recovered stress and strain at break cannot be the result of humins post-condensation and subsequent Diels–Alder crosslinking is necessary to achieve the reported healing efficiencies. Similar approaches have been proposed for thermosets by the incorporation of thermoplastic polymers in blended [52] or semi-interpenetrated [53] polymer network systems. Upon heating above the thermal transition of the thermoplastic, the linear polymer flows into the crack, sealing the damage and recovering the mechanical properties upon solidification during cooling. To the authors’ knowledge, this is the first time that a similar principle is reported for elastomeric intrinsic self-healing polymers using an aiding mechanism based on a mobile agent under mild conditions. Thus, humins are a promising candidate to both enhance the healing performance of conventional thermoreversible SH materials and lower their cost by means of valorizing a biorefinery waste.

### 3.5. Humins-DA Reprocessability

One of the greatest advantages that thermoreversible polymer networks offer is the combination of excellent mechanical strength as seen in traditional elastomers and their thermal reprocessability, by heating above the reversible gel transition temperature T_gel_ or dissolving in a suitable solvent. This further promotes the sustainability of thermoreversible covalent networks besides their SH performance [44]. If the SH material is submitted to a too aggressive, unhealable damage (e.g., a robotic actuator working in a harsh environment) the material loses its functionality and needs to be replaced. A few approaches have been explored in literature to overcome large damage issues on SH materials, such as magnetically driven damage closure [51] or shape-memory assisted self-healing (SMASH) [53,54]. Unless SH materials have these added functionalities, reprocessing is the only valid route to revalorize the materials after unrepairable damage. In this research, it was observed that Humins-DA was extrudable at 100 °C, i.e., at a temperature well below T_gel_ (see Figure 3b), which highlights the possibility to (re)process the material at low enough temperatures during short processing timescales to limit the influence of humins post-condensation reactions on the properties to a minimum. The fact that the material could be extruded at temperatures below T_gel_ was due to the well-differentiated flow behavior of the composite components and the reversible dynamics of the Diels–Alder reaction. On the one hand, the conversion of Diels–Alder-based polymer networks was above the critical gel conversion at temperatures below T_gel_, resulting in a crosslink density high enough to keep the gel structure at such temperatures. On the other hand, the reversible dynamics of the Diels–Alder reaction increased significantly and the viscosity of the humins decreased strongly with temperature (Figure 1f). Furthermore, upon high shear stress, it is not unlikely that reversible bonds are further broken during extrusion. The overall behavior is translated into a gel with low enough (visco)elastic properties and high enough dynamic reactivity, making it suitable to be manufactured under the designed conditions.

**Figure 6:**
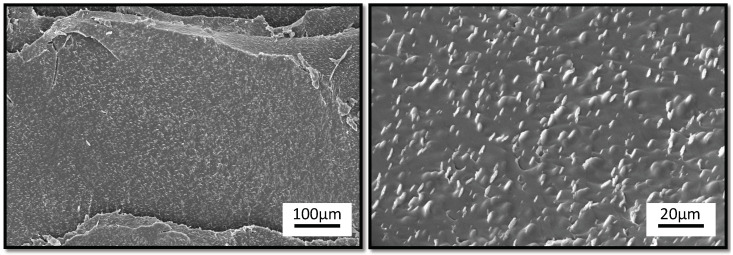
SEM images of the extruded Humins-DA cross-section.

Reprocessing also influences the polymer blend microstructure (Figure 6). Some authors have reported DA-based polymer blends, observing nanosized droplets-in-matrix morphologies strongly influenced by the applied thermal treatments [55]. Intense mixing under high shear forces in the twin screw extruder has resulted in a much finer and homogeneous droplet morphology of the humins in the DA matrix (2–6 μm diameter). The fact that humins morphology can be controlled under shear flow in an immiscible blend allows a plethora of potential opportunities to tailor their morphology, and hence, the final blend properties. The mechanical properties of the extruded Humins-DA were assessed before and after healing under different conditions (Table 4). First, it should be noted that the mechanical properties of the extruded specimens were higher than the untreated blend, undergoing a stiffening as also seen for the thermally treated samples (Table 3). The Young’s modulus after extrusion at 100 °C was between the moduli of the blends that were thermally treated at 90 °C for 1 and 2 h. The fracture strain was similar to the thermally treated samples, while the stress at fracture was significantly higher than all thermally treated samples. Similar to the thermally treated samples, the healing efficiency after 1 h at 60 °C reduced compared to the untreated material, showing a considerable improvement by increasing the healing temperature to 80 °C. The healed samples showed a pronounced further stiffening with an increased Young’s modulus. The healing performance of the extruded samples after 1 h at 60 °C was lower than that of the non-extruded, thermally treated counterparts, while the healing efficiency at 80 °C for the extruded samples fell between those of the samples that were thermally treated at 90 °C for 1 and 2 h. From an application point of view, it is important to consider this aspect, as it can be assumed that the healing properties decrease after several reprocessing cycles, similarly to the annealed samples.

Two potential explanations are given to elucidate these properties variations when comparing to the non-extruded Humins-DA. On the one hand, a lower healing efficiency can be explained by a reduction of the droplet size of humins. Smaller droplets, combined with a high interfacial tension, hinders humins flow out of the matrix confinement, thus limiting their influence in the healing mechanism. This confirms that the humins droplets morphology has an important influence on the healing mechanism, especially enabling healing at lower temperatures, by the hypothesized wetting of the damage surfaces and aiding the broken chain rearrangement and, hence, DA bonds reformation. On the other hand, considering the mechanical properties, two potential explanations could enlighten the observed variations. First, the short but highly energetic heating step performed during extrusion, together with the high applied shear forces, can induce a thermo-mechanochemical response on humins curing that accelerates their crosslinking reaction kinetics even at low timescales. Approaches such as on-line ATR-FTIR monitoring of polymer melts at the extruder barrel have been previously explored, showing promising potential applicability in the study of humins behavior during processing [56]. Second, smaller humins droplets confined within the DA matrix lead to a higher interfacial surface area. A faster gelation due to the DA higher conversion at the extrusion temperature (a gel is still present) followed by enhanced DA reaction kinetics at high temperatures, hinders humins droplets nucleation and growth. Thus, a finer humins droplet-matrix phase dispersion is achieved. As a consequence, a better dispersed phase can more efficiently distribute the applied load force, thus increasing the final mechanical performance.

### 3.6. Soft Robotic Gripper as a Multi-Material Demonstrator

**Figure 7:**
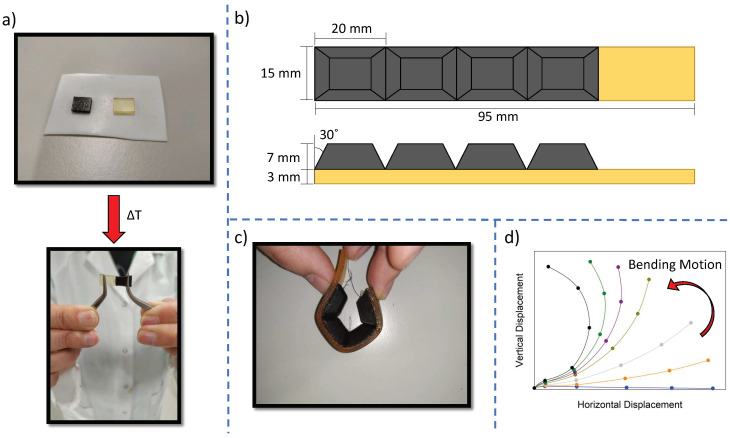
(**a**) Multi-material connectivity between BMI689-F400 (yellow) and Humins-DA (black) by healing two pieces together at 60 °C for 1 h. (**b**) Schematic representation of the multi-material robotic finger assembly. (**c**) Robotic multi-material finger built showing high flexibility. (**d**) Bending tendon-driven motion test of the prepared robotic finger. The blue curve corresponds to the finger position at rest, whereas the black curve corresponds to the final bent position. The rest of the curves, that is, orange, grey, dark yellow, purple and green, describe the finger transition from the position at rest to the bent state.

To explore the potential in an end-use application, the Humins-DA system was exploited in a soft robotic demonstrator. The high loading of the liquid humins strongly affects the elastic recovery of the material due to viscous losses. To overcome this issue, authors have reported the assembly of multi-material robotic structures combining two different samples with different mechanical properties, so that a more elastic phase can take care of the mechanical compliance while a less compliant but softer phase interacts with the environment, being easily healed under mild conditions [57]. The dynamic covalent bonds are also active at the interface, so the materials can be strongly fused together upon thermal treatment [30]. To manufacture the soft finger, a thermal pre-treatment was first applied on BMI689-F400 at 110 °C for 10 min to reduce the crosslinking concentration and obtain enough free furan and maleimide moieties at the sample surface for an efficient binding. Upon BMI689-F400 furan and maleimide moieties activation, the material fragments were brought in contact with a piece of Humins-DA and kept at 60 °C for 1 h to promote the (re)formation of DA interfacial bonds. A temperature of 60 °C was used to try to keep Humins-DA integrity and limit thermal stiffening to the greatest extent while an efficient fusion on the interface is obtained. As can be observed in Figure 7a, a good interfacial contact was achieved under the proposed conditions for a multi-material assembly. Following the same procedure, a series of multi-material robotic fingers were created using the same method as Roels et al. to build a gripper (Figure 7b) [57]. The resulting assembled finger consisted of a BMI689-F400 base (yellow) fused to four Humins-DA phalanges (black), exhibiting an excellent interfacial connectivity and part flexibility as shown in Figure 7c. A hole was drilled in each phalanx through which the tendon cable (nylon fishing wire) was routed. The holes were lined with Teflon tubing to avoid the tendon cutting into the soft material. The bending of the finger was characterized using the test setup as described in Section 2.4. The flexing motion was achieved by pulling the tendon cable, and the finger returned to the initial position by recovering the elastic energy stored in the material when the tendon was released. The finger actuation performance is presented in Figure 7d, observing a suitable bending motion for tendon-driven grasping actuation. The maximal achieved bending angle was 84°, measured as the angle between the horizontal axis and the line connecting base to tip. A common mode of failure in soft robotics is debonding of multi-material parts at the interface due to a lack of strong interfacial bonds. The use of self-healing polymers overcomes this problem.

**Figure 8:**
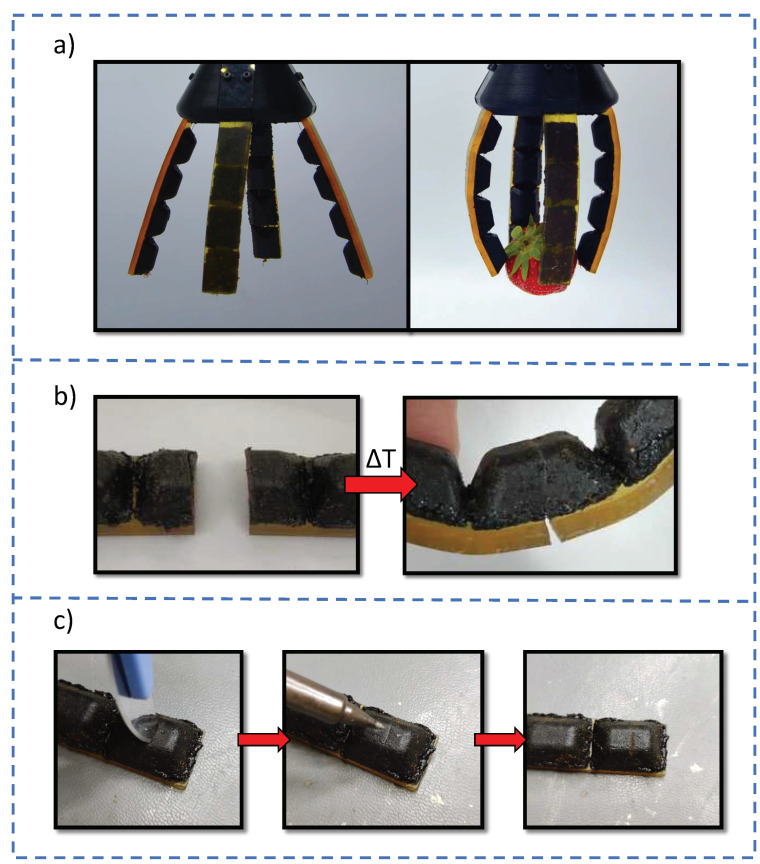
(**a**) Multi-material humins-based soft gripper grabbing a soft object. (**b**) Fractured multi-material finger showing great connectivity of the Humins-DA component under a healing treatment of 60 °C for 1 h. (**c**) Focalized healing-by-welding of Humins-DA upon damage at 100 °C for a few seconds.

Four fingers were assembled in a square pattern to form a soft gripper that is able to pick up objects of different size and shape. As can be observed in Figure 8a, even delicate objects can be manipulated without damaging. To corroborate the improved healing performance of Humins-DA compared to the BMI689-F400, the finger was cut in half using a scalpel blade. Subsequently, the broken parts were put back in contact and healed at 60 °C for 1 h. Figure 8b shows how the Humins-DA component exhibited an excellent healing performance, whereas the BMI689-F400 broken parts remained separated, confirming previous observations.

Global heating is undesired for real applicability since each healing cycle entails modifications on Humins-DA mechanical properties due to humins crosslinking. To overcome this issue, a hot tip welder heated up to 100 °C was used to execute a localized damage healing. Using this approach, not only was the damage successfully closed upon contacting for a few seconds, but this can also be used to reinforce the damaged zone by means of humins crosslinking, preventing future potential damage at the healed zone (see Figure 8c). To automatize the healing process and make it more efficient, other methodologies such as optothermal healing could be considered for longer and more precise thermal exposures. Laser sources have already been proven as a suitable technique to perform localized healing thermal treatments [58,59]. Inspired by biology, this behavior resembles soft tissues able to grow calluses when subjected to aggressive mechanical friction or pressure, generating extra skin layers for protective functions. This research shows, for the first time, the formulation of an intrinsic self-healable system that can both heal and reinforce the heated zone, finding a promising suitability in the sustainable soft robotics field [31].

## 4. Conclusions

Humins formation is a biorefinery issue of great concern; thus, new routes towards their valorization are needed to prevent waste formation and accumulation and to pave a path to improve the economic viability of their processes. In this research, it was demonstrated that humins blended into a reversible covalent polymer network facilitate the self-healing behavior under the proposed compositions and healing conditions. For classical furan-maleimide Diels–Alder systems, an energetic thermal treatment needs to be applied to unbind a sufficient amount of reversible covalent bonds to have a sufficiently high mobility for an efficient and effective healing process. It was shown that the humins enable healing of fatal damage and successful recovery of the mechanical properties at temperatures of 60 and 80 °C, where the pristine DA polymer network does not have the required reactivity and mobility for damage healing. The facilitation of the healing behavior by the humins is a result of physical processes aiding the subsequent reformation of Diels–Alder bonds as is well established for such self-healing materials. While partially phase separated, the humins provide a plasticizing effect on the polymeric network, improving the polymer chain mobility and creating an effective contact between the damage surfaces. The strong change in viscosity of the humins upon mild heating further facilitates the healing action at moderate temperatures of around 60 °C. Thus, lower temperatures are required to achieve an efficient mended state while keeping high mechanical properties compared to other reported DA networks. Moreover, the addition of a large fraction of humins improves the ecological and economic impact of the final self-healing blend by valorizing the humins waste stream. 

Thermal crosslinking of humins is detected during healing at more elevated temperatures. On the one hand, this poses a limit to the number of thermal healing cycles and thermal reprocessing cycles that can be performed. On the other hand, this can be exploited for the preparation of stiffness-tunable humins-based self-healable materials upon thermal treatment, which possess a higher stiffness than the raw DA material but also a better healing performance. It has been demonstrated that Humins-DA is a suitable and sustainable solution for the preparation of novel soft robotic systems that can be healed locally to recover their actuation performance while achieving a reinforced state, thus reducing any potential future damage at the healed zone. This approach mirrors for the first time the formation of protective calluses existing in biological systems on an artificial intrinsic self-healable composite. 

It should be noted that under mild healing conditions, e.g., at 60 °C, the stiffening effect was negligible, while the healing was also slower at these temperatures. At moderate temperatures, the contribution of the humins to the self-healing performance was purely of a physical nature and was thus an aiding mechanism to facilitate the healing by way of the reformation of Diels–Alder bonds. At more elevated temperatures, due to the crosslinking of the humins, the humins actively participated in the reformation of (additional) covalent crosslinks in a chemical manner, in addition to the physical facilitation.

## Figures and Tables

**Table 1:** Peaks assignments of humins FT-IR spectra.

Wavenumber (cm^−1^)	Peak Assignments
3370	O–H stretching
3127	(C=C)–H stretching
2926, 2829	C–H (sp^3^) stretching
1701	C=O stretching
1671	C=O stretching (Furan)
1608, 1515	C=C stretching
1393, 1359	O–H bending
1281, 1183, 1076, 1017	C–O stretching C–O stretching (furan)
895	C=C bending
812–749	C–H bending out of plane

**Table 2:** Summary of the mechanical properties (fracture stress, fracture strain, and Young’s modulus) measured for BMI689-F400 and Humins-DA, together with their respective healing efficiencies.

Sample		σ (MPa)	η_σ_ (%)	ε (%)	η_ε_ (%)	E (MPa)	η_E_ (%)
BMI689-F400	Initial	1.86 ± 0.14	0	141 ± 22	0	3.65 ± 0.20	0
	Healed 1 h 60 °C	Not Healed		Not Healed		Not Healed	
Humins-DA	Initial	0.30 ± 0.11	82	134 ± 36	78	2.07 ± 0.40	177 *
	Healed 1 h 60 °C	0.24 ± 0.04		104 ± 17		3.67 ± 0.73	

*: Non-representative result as a consequence of humins crosslinking reactions.

**Table 3:** DMA parameters obtained for the annealed Humins-DA, together with the respective healing efficiencies at both 60 °C and 80 °C for 1 h.

Sample		σ (MPa)	η_σ_ (%)	ε (%)	η_ε_ (%)	E (MPa)	η_E_ (%)
Humins-DA 90 °C 1 h	Initial	0.29 ± 0.03	-	110 ± 20	-	2.47 ± 0.21	-
	Healed 1 h 60 °C	0.21 ± 0.09	71	34 ± 15	31	4.27 ± 0.38	172 *
	Healed 1 h 80 °C	0.54 ± 0.17	187 *	93 ± 24	84	9.06 ± 1.23	366 *
Humins-DA 90 °C 2 h	Initial	0.37 ± 0.01	-	119 ± 26	-	4.27 ± 0.62	-
	Healed 1 h 60 °C	0.29 ± 0.06	77	35 ± 8	30	5.80 ± 0.45	136 *
	Healed 1 h 80 °C	0.33 ± 0.10	89	36 ± 9	30	4.60 ± 0.20	108 *
Humins-DA 90 °C 3 h	Initial	0.61 ± 0.08	-	98 ± 11	-	6.84 ± 0.93	-
	Healed 1 h 60 °C	0.25 ± 0.06	40	19 ± 5	19	6.49 ± 1.18	95
	Healed 1 h 80 °C	0.43 ± 0.10	70	31 ± 13	32	8.48 ± 3.19	124 *

*: Non-representative results as a consequence of humins crosslinking reactions.

**Table 4:** DMA parameters obtained for extruded Humins-DA, together with the respective healing efficiencies at both 60 and 80 °C for 1 h.

Sample		σ (MPa)	η_σ_ (%)	ε (%)	η_ε_ (%)	E (MPa)	η_E_ (%)
Extruded Humins-DA	Initial	0.77 ± 0.07	-	111 ± 18	-	3.40 ± 0.18	-
	Healed 1 h 60 °C	0.36 ± 0.09	47	27 ± 4	24	5.00 ± 0.04	147 *
	Healed 1 h 80 °C	0.68 ± 0.17	88	63 ± 22	56	5.33 ± 1.05	157 *

*: Non-representative result as a consequence of humins crosslinking reactions.

## Data Availability

Not applicable.

## Supplementary Materials

The following supporting information can be downloaded at: https://www.mdpi.com/article/10.3390/polym14091657/s1, Figure S1: FT-IR spectra of raw BMI689-F400 (red line) and BMI689-F400 substracted from the Humins-DA spectra (black line); Figure S2: SEM picture of the Humins-DA crossection thermally treated at 80 °C for 2 h.
