# Supplementary material for: Humins Blending in Thermoreversible Diels–Alder Networks for Stiffness Tuning and Enhanced Healing Performance for Soft Robotics
Source: Polymers (Basel). 2022 Apr 20;14(9):1657. doi: 10.3390/polym14091657 (PMC9101211; doi:10.3390/polym14091657)
Supplement: Supplementary file 1 [file polymers-14-01657-s001.zip › polymers-1669777-supplementary.pdf]

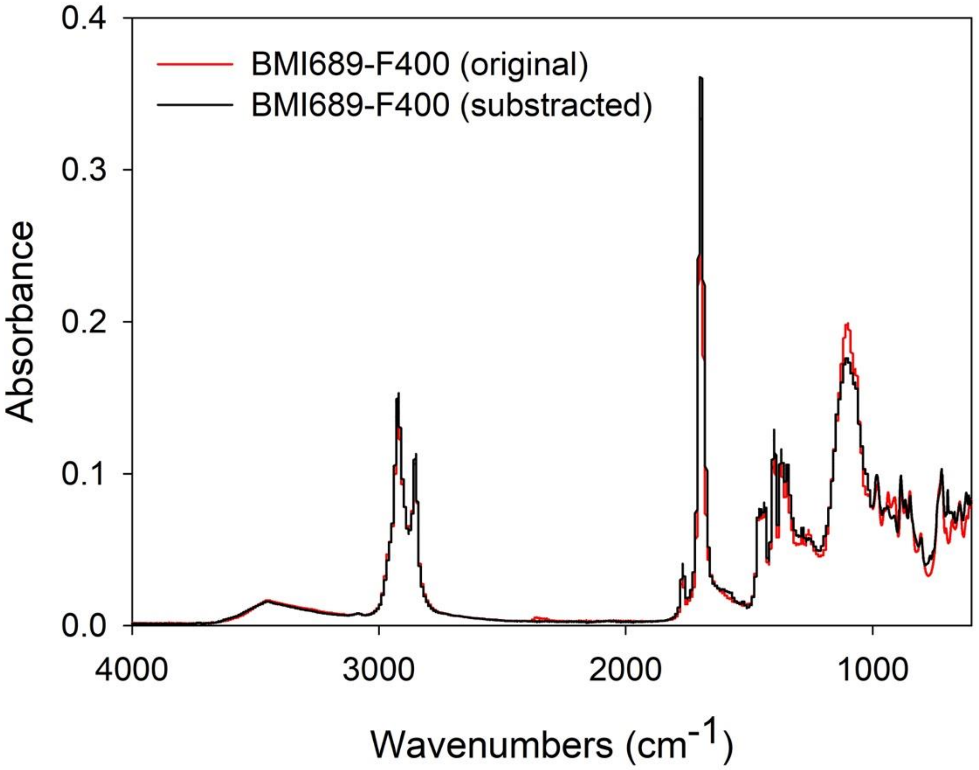

Figure S1. FT-IR spectra of raw BMI689-F400 (red line) and BMI689-F400 substracted from the Humins-DA spectra (black line).

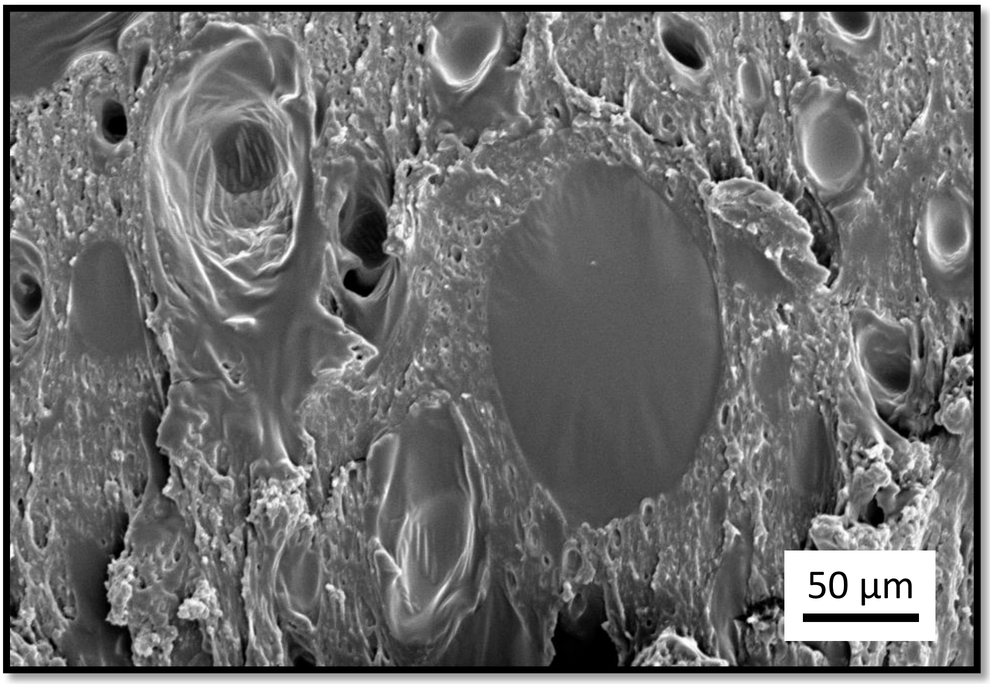

Figure S2. SEM picture of the Humins-DA crossection thermally treated at 80˚C for 2 hours.
